# Open Versus Laparoscopic Donor Nephrectomy in Pediatric Kidney Transplantation: A Systematic Review and Meta-Analysis

**DOI:** 10.3390/jcm15197484

**Published:** 2026-09-26

**Authors:** Rajan Nikbakhsh, Sepehr Abbasi Dezfouli, Nastaran Sabetkish, Mohammadamin Shahrbaf, Yalda Mirzaei, Saad Hifdi, Mohammadsadegh Sabagh, Peri Husen, Georg Lurje, Claus Peter Schmitt, Burkhard Tönshoff, Arianeb Mehrabi, Elias Khajeh

**Affiliations:** 1 Department of General, Visceral and Transplantation Surgery, Heidelberg University, 69120 Heidelberg, Germany; rajan.nikbakhsh@med.uni-heidelberg.de (R.N.); sepehrabbasi@gmail.com (S.A.D.); mohammadsadegh.sabagh@bgu-ludwigshafen.de (M.S.);; 2 Institute for Laboratory Animal Science, Unklinik RWTH Aachen, 52074 Aachen, Germany; 3 Department of Paediatrics I, Centre for Pediatric and Adolescent Medicine, Medical Faculty Heidelberg, Heidelberg University, 69120 Heidelberg, Germany; 4 Department of Pediatrics I, University Children’s Hospital Heidelberg, 69120 Heidelberg, Germany; burkhard.toenshoff@med.uni-heidelberg.de

**Keywords:** pediatric kidney transplantation, living donors, laparoscopic nephrectomy, open nephrectomy

## Abstract

**Background/Objectives**: Although minimally invasive laparoscopic donor nephrectomy (LDN) has been established as the standard method for donor nephrectomy for adult recipients in recent years, there might still be a role for conventional open donor nephrectomy (ODN) in pediatric kidney transplantation (KTx). To investigate this, we compared the outcomes of LDN with those of ODN in pediatric patients. **Methods**: A systematic literature search was conducted in Web of Science and Medline (via PubMed) databases, focusing on intraoperative data in donors and short-term postoperative outcomes in pediatric recipients following ODN and LDN. We used a subgroup analysis to compare these outcomes in recipients aged five years or younger. **Results**: Fifteen datasets comprising 1926 pediatric kidney transplant recipients (1036 ODN and 890 LDN) were included in the quantitative synthesis. There were no significant differences in operation time, warm ischemia time, or blood loss among donors (*p* > 0.05). There were also no significant differences in the rates of acute graft rejection, delayed graft function, or one-year graft survival between pediatric recipients undergoing ODN and LDN. In the subgroup analysis, the rates of delayed graft function and acute graft rejection were significantly lower in recipients aged ≤5 years in the ODN group compared with the LDN group (Odds ratio (OR), 0.26; *p* = 0.041 and OR, 0.29; *p* = 0.016, respectively). The certainty of evidence was very low across all outcomes according to the GRADE assessment. **Conclusions**: Our findings suggest that outcomes following ODN and LDN appear broadly comparable in pediatric kidney transplantation. However, as the available comparative evidence is derived primarily from studies conducted during the early adoption era of laparoscopic donor nephrectomy, these findings should be interpreted cautiously and mainly in a historical context. The overall certainty of evidence was rated as very low according to GRADE. Contemporary data from high-volume transplant centers are needed to better define outcomes in very young pediatric recipients.

## 1. Introduction

Kidney transplantation (KTx), particularly preemptive transplantation, is the treatment of choice for children with end-stage kidney disease (ESKD), providing substantial advantages in survival, growth, development, and quality of life compared with long-term dialysis [1]. However, pediatric KTx presents specific technical and physiological challenges that distinguish it from transplantation in adults. These challenges are particularly pronounced in very young recipients, in whom small-caliber vessels, limited intra-abdominal space, donor–recipient size mismatch, and greater sensitivity to perioperative hemodynamic changes may increase the complexity of transplantation and the risk of early graft complications [2]. Accordingly, the surgical approach to pediatric KTx is strongly dependent on recipient size, with very small children often requiring intraperitoneal placement of an adult-sized graft and different vascular anastomotic strategies than larger children and adults [2]. Such donor–recipient size disparities also pose distinct hemodynamic challenges during graft reperfusion, while differences in immune responsiveness between pediatric and adult recipients may further influence post-transplant outcomes [3]. Vascular thrombosis represents an additional concern in small pediatric recipients and remains an important cause of early graft loss [2,4]. Moreover, graft outcomes in children may be influenced by recipient hemodynamics, fluid management, donor–recipient size matching, and center-specific surgical and perioperative expertise [5,6]. Consequently, evidence derived from adult kidney transplantation cannot necessarily be extrapolated directly to very young pediatric recipients [3].

Living-donor kidneys can be procured using either open donor nephrectomy (ODN) or laparoscopic donor nephrectomy (LDN). Since its introduction, LDN has become the preferred approach in many transplant centers, primarily because of its donor-centered benefits, including reduced surgical trauma and postoperative pain, shorter hospital stay and recovery time, and improved cosmetic outcomes [7,8]. However, these advantages for the donor should be distinguished from potential graft- and recipient-related implications of the procurement technique. LDN requires advanced laparoscopic expertise and may be associated with longer warm ischemia time, particularly during the surgical learning curve [7,8]. Furthermore, the pneumoperitoneum required during laparoscopic procurement can transiently alter renal hemodynamics and reduce renal perfusion before graft retrieval [3]. Whether these technical and physiological differences translate into clinically relevant effects on early graft function or recipient outcomes, including delayed graft function, acute graft rejection, and vascular thrombosis, remains uncertain, particularly in very young pediatric recipients [9,10].

The available comparative evidence is nevertheless limited. Most studies are retrospective, include relatively small pediatric cohorts, and originate predominantly from the early adoption era of LDN, when surgical experience, instrumentation, and perioperative management differed from contemporary practice. In addition, differences in recipient characteristics, donor selection, surgical expertise, and outcome definitions contribute to clinical and methodological heterogeneity across studies. These limitations leave uncertainty regarding whether the donor nephrectomy approach has clinically relevant implications for pediatric recipients, particularly for very young children. We therefore conducted a systematic review and meta-analysis comparing donor and recipient outcomes following ODN and LDN in pediatric KTx. Given the distinct technical and physiological challenges encountered in very small recipients and previous evidence suggesting that hemodynamic and graft-related vulnerabilities may vary with recipient age [3], we prespecified a subgroup analysis of recipients aged ≤5 years to evaluate whether outcomes associated with donor nephrectomy approach differed in this particularly vulnerable population.

## 2. Materials and Methods

The present meta-analysis was conducted according to the Preferred Reporting Items for Systematic Reviews and Meta-Analyses (PRISMA) 2020 guidelines [11] and Study Center of the German Society of Surgery recommendations [12]. The protocol was registered in the International Prospective Register of Systematic Reviews with the registration code CRD4202450558. The completed PRISMA 2020 checklist is provided in Supplementary Table S5.

### 2.1. Search Strategy and Selection Criteria

The study question was formulated using the Population, Intervention, Comparison, Outcome, and Study design (PICOS) strategy:

- **Population:** pediatric recipients who underwent a living-donor kidney transplant.
- **Intervention: Ldn.**
- **Comparator: Odn.**
- **Outcome:** Perioperative outcomes, delayed graft function (defined as need for dialysis within the first week after KTx), acute graft rejection (defined as treatment for rejection within the first six months after KTx), and one-year graft survival.
- **Study design**: All studies comparing ODN and LDN in a pediatric population, except case reports, case series with fewer than 10 cases, editorials, and letters to the editor.

The systematic literature search was conducted in MEDLINE via PubMed and Web of Science through 31 December 2025. No language restrictions were applied during the literature search or study selection process. In addition, the reference lists of included studies and relevant review articles were manually screened to identify additional potentially eligible studies.

Three investigators (RN, NS, and SH) conducted the literature search. The complete database-specific search strategies for MEDLINE via PubMed and Web of Science are provided in Supplementary Text S1.

First, the titles and abstracts of the retrieved articles were independently screened by two investigators (R.N. and N.S.) according to the predefined inclusion and exclusion criteria. Subsequently, the full texts of potentially eligible studies were independently assessed by the same two investigators. Any disagreements were resolved through consultation with the corresponding author (E.K.).

### 2.2. Data Extraction and Risk of Bias

Demographic characteristics of the included studies, including publication year, country, number of patients in each group, and participant age and sex, were collected. Intraoperative variables, including operation time, estimated blood loss, and warm ischemia time, as well as postoperative recipient outcomes, including acute graft rejection, delayed graft function, and one-year graft survival, were extracted and recorded in an Excel datasheet. Data extraction was performed independently by three authors, and any discrepancies were resolved by consensus.

Warm ischemia time (WIT) was analyzed separately for donors and recipients. Donor WIT was defined according to the definitions reported in the individual studies and generally represented the interval between interruption of renal blood flow during donor nephrectomy and initiation of cold preservation. Recipient WIT represented the period during transplantation from removal of the graft from cold preservation until reperfusion following completion of the vascular anastomoses. Because the exact definitions and measurement methods were not uniformly reported across studies, potential between-study variation in WIT definitions was considered when interpreting the pooled estimates.

In addition to the 14 studies identified through the systematic database search, data from our institutional cohort at Heidelberg University Hospital were incorporated separately into the quantitative synthesis. The underlying pediatric kidney transplantation cohort has previously been published by Mehrabi et al. (2019) [13], whereas the specific comparative analysis of ODN versus LDN included in the present study represents a subsequent analysis of the institutional data. The institutional study was approved by the Ethics Committee of the Medical Faculty of Heidelberg University (approval number S-811/2018) and was conducted in accordance with the Declaration of Helsinki. The requirement for study-specific informed consent was waived; however, prior to transplantation, all patients or their legal guardians had provided consent for the use of their clinical data for scientific research. The institutional dataset fulfilled the same eligibility criteria and was assessed using the same outcome definitions, risk-of-bias assessment, and statistical methodology as the studies identified through the systematic search.

Risk of bias was assessed independently by two authors (R.N. and N.S.) using the Risk Of Bias In Non-randomized Studies of Interventions (ROBINS-I) tool Version 2. Any disagreements were resolved by consensus.

### 2.3. Quality Assessment and Risk of Bias

The risk of bias was assessed using the Risk Of Bias In Non-randomized Studies of Interventions (ROBINS-I) tool [14]. The overall certainty of evidence for the evaluated outcomes was assessed using the Grading of Recommendations Assessment, Development and Evaluation (GRADE) approach [15].

### 2.4. Statistical Analysis

To analyze the data, R software version 4.2.1 (R Foundation for Statistical Computing, Vienna, Austria) was utilized. Variables were reported using a random-effects model. Effect sizes were presented as odds ratios (ORs) for dichotomous data and mean differences (MDs) for continuous data, both with 95% confidence intervals (CIs). Statistical heterogeneity was evaluated using the χ^2^ test and inconsistency analyses, with significance defined as a *p* value < 0.05 and an I^2^ value exceeding 50%. If significant heterogeneity was detected, a sensitivity analysis employing the leave-one-out method was performed [16].

## 3. Results

### 3.1. Review of the Literature and Characteristics of Selected Articles

The systematic literature search initially identified 1077 records. After removal of duplicates, 912 records were screened based on their titles and abstracts. Of these, 96 full-text reports were assessed for eligibility, and 14 studies identified through the systematic search were included in the quantitative synthesis (Figure 1). In addition, data from our previously published institutional cohort (Mehrabi et al., 2019 [13]) were incorporated into the quantitative synthesis, resulting in a total of 15 studies. The reasons for exclusion are explained in Figure 1. These 15 studies included a total of 1926 recipients (1036 in the ODN group and 890 in the LDN group). Preemptive transplantation was reported in nine studies (10–45%), and re-transplantation was recorded in four studies (2–8%). These data are summarized in Table 1.

### 3.2. Risk of Bias Assessment in Included Studies

Fifteen retrospective datasets were assessed for risk of bias using the ROBINS-I tool. Six were judged to have a serious overall risk of bias and nine a moderate risk of bias. The main sources of bias were related to participant selection, confounding, and outcome measurement. Detailed domain-level ROBINS-I judgments for each included dataset are provided in Supplementary Table S1. These methodological limitations were considered when interpreting the pooled estimates and contributed to downgrading the certainty of evidence. The GRADE assessment, including outcome-specific judgments for risk of bias, inconsistency, indirectness, imprecision, and publication bias, is provided in Supplementary Table S2. Overall, the certainty of evidence was rated as very low across the assessed outcomes, and the pooled findings were therefore interpreted cautiously.

### 3.3. Intraoperative Donor Surgical Outcomes

#### 3.3.1. Operation Time

Six studies compared operation time between ODN (n = 186; mean ± SD, 189.67 min ± 83.01) and LDN (n = 108; mean ± SD, 203.49 min ± 60.57). The random-effects model showed no significant difference between these groups (*p* = 0.938; MD, 1.69; 95% CI, −41.27 to 44.65; Figure 2). Pooled studies were heterogeneous (I^2^ = 94.5%; *p* < 0.0001). However, leave-one-out method of sensitivity analysis showed robustness of the previous outcome.

#### 3.3.2. Estimated Blood Loss

Four studies were included in the quantitative analysis of estimated blood loss. A random-effects model showed no significant difference in estimated blood loss between the ODN group (n = 134; mean ± SD, 122.18 mL ± 122.56) and the LDN group (n = 83; mean ± SD, 113.90 mL ± 121.77) (*p* = 0.328; MD, 35.09; 95% CI, −35.26 to 105.43; Figure 3). The included studies were heterogeneous (I^2^ = 83%, *p* = 0.0005). Sensitivity analysis excluding Singer 2005 [21] substantially altered the pooled estimate. After removal of this study, estimated blood loss was significantly higher in the ODN group compared to the LDN group (MD 73.39 mL, 95% CI 32.67 to 114.11; *p* = 0.0004). Between-study heterogeneity markedly decreased (I^2^ = 33.6%, *p* = 0.222), indicating that Singer 2005 [21] was a major contributor to heterogeneity and influenced the overall effect estimate.

#### 3.3.3. Warm Ischemia Time

The warm ischemia time was analyzed in six studies. Two studies comprising 94 donors reported donor warm ischemia time: 59 in the ODN group (mean ± SD, 4.16 min ± 1.23) and 35 in the LDN group (mean ± SD, 6.56 min ± 1.84). The final random-effects model demonstrated no statistically significant difference between these two groups (*p* = 0.060; MD, −2.58; 95% CI, −5.28 to 0.11; Figure 4A). Significant heterogeneity was observed between the two studies (I^2^ = 94.5%; *p* < 0.0001). Four studies comprising 187 recipients reported recipient warm ischemia time: 124 in the ODN group (mean ± SD, 33.67 min ± 19.32) and 63 in the LDN group (mean ± SD, 39.10 min ± 15.08). The final random-effects model demonstrated no statistically significant difference between these two groups (*p* = 0.322; MD, −4.23; 95% CI, −12.63 to 4.16; Figure 4B). There was significant heterogeneity between the four included studies (I^2^ = 86.7%; *p* < 0.0001). However, leave-one-out method of sensitivity analysis showed robustness of the previous outcome.

### 3.4. Post-Ktx Outcomes in Pediatric Recipients

Study-level sample sizes and raw event data for delayed graft function, acute graft rejection, one-year graft survival, and vascular thrombosis are provided in Supplementary Table S4.

#### 3.4.1. Delayed Graft Function

Eleven studies were included in the quantitative analysis of delayed graft function, comprising 1673 recipients. Of these, 859 underwent ODN and 814 underwent LDN. A random-effects model demonstrated no significant difference in the rate of delayed graft function between the ODN group (44/859) and the LDN group (56/814) (*p* = 0.468; OR, 0.81; 95% CI, 0.42 to 1.53; Figure 5A). No heterogeneity was observed between the pooled studies (I^2^ = 33%; *p* = 0.12).

Four studies were included in the subgroup analysis of pediatric recipients aged five years or younger. This analysis showed a significantly lower rate of delayed graft function in the ODN group than in the LDN group (*p* = 0.041; OR, 0.26; 95% CI, 0.07 to 0.91; Figure 5B). There was no heterogeneity between the included studies (I^2^= 0%; *p* = 0.78).

#### 3.4.2. Acute Graft Rejection

Acute graft rejection was evaluated in eleven studies, including 932 patients who underwent ODN and 816 patients who underwent LDN. A random-effects model demonstrated no significant difference in rejection rate between LDN (196/816 recipients) and ODN group (159/932 recipients) (*p* = 0.132; OR, 1.45; 95% CI, 0.87 to 2.42; Figure 6A). There was significant heterogeneity between these studies (I^2^ = 63%; *p* = 0.0024).

The subgroup analysis of recipients aged five years or younger included four studies and showed a significantly lower acute graft rejection rate in the ODN group than in the LDN group (*p* = 0.016; OR, 0.29; 95% CI, 0.13 to 0.65; Figure 6B). There was no heterogeneity between these four studies (I^2^ = 0%; *p* = 0.77).

#### 3.4.3. One-Year Graft Survival

One-year graft survival was analyzed in seven studies, including 896 recipients who received ODN and 735 who received LDN. The random-effects model found no significant difference in one-year survival between the ODN group (850/896) and the LDN group (701/735) (*p* = 0.237; OR, 0.58; 95% CI, 0.20 to 1.65; Figure 7). No heterogeneity was observed between these studies (I^2^ = 50%; *p* = 0.07). Separate outcomes in younger recipients were not reported in the included studies.

#### 3.4.4. Vascular Thrombosis

Vascular thrombosis was evaluated in four studies, including 689 patients who underwent ODN and 558 patients who underwent LDN. A random-effects model demonstrated no significant difference in the rate of vascular thrombosis between LDN (14/558 recipients) and ODN group (15/689 recipients) (*p* = 0.115; OR, 0.46; 95% CI, 0.15 to 1.41; Figure 8). There was no heterogeneity between these four studies (I^2^ = 0%; *p* = 0.55).

## 4. Discussion

Kidney transplantation in very young pediatric recipients represents a highly complex surgical and multidisciplinary challenge. Technical factors such as small-caliber vessels, donor–recipient size mismatch, and limited intra-abdominal space increase the risk of perioperative complications, particularly vascular thrombosis, which remains one of the leading causes of early graft loss in this population [4]. In addition, graft outcomes are strongly influenced by recipient hemodynamics, perioperative fluid management, anesthetic strategies, and institutional expertise [5]. Previous studies have demonstrated substantial variability between pediatric transplant centers regarding intraoperative monitoring, blood pressure targets, fluid administration, and postoperative management protocols, highlighting the absence of a standardized perioperative approach [5]. Furthermore, donor–recipient body surface area mismatch has been shown to significantly affect long-term graft survival, emphasizing the importance of careful donor selection in pediatric recipients [6]. Therefore, outcomes in pediatric kidney transplantation are likely determined by multiple interrelated surgical and perioperative factors rather than donor nephrectomy technique alone. This may partly explain why the role of ODN versus LDN remains difficult to define in contemporary practice and is likely highly dependent on center experience and multidisciplinary expertise.

Another challenge in pediatric KTx is achieving the long-term graft survival needed to maintain the life expectancy of young recipients. In particular, small children have a higher risk of surgical complications after KTx [28]. Although differences in warm ischemia time between ODN and LDN have raised concerns regarding potential effects on graft outcomes [13], our overall pooled analyses demonstrated no significant differences in delayed graft function, acute graft rejection, or one-year graft survival between the two approaches. Therefore, the available evidence does not demonstrate a general recipient-related advantage of ODN over LDN in the overall pediatric population.

We found no significant differences in the incidence of acute graft rejection and delayed graft function between pediatric patients undergoing ODN and LDN. However, our subgroup analysis in patients aged five years or younger revealed a higher rate of acute graft rejection and delayed graft function following LDN than following ODN—a finding that is consistent with United Network for Organ Sharing (UNOS) analysis [29]. Abrahams et al. showed excellent organ function after LDN in pediatric recipients; this organ function was similar to that observed after traditional ODN [19]. Similarly, Hsu et al. reported excellent 24-month graft function after LDN in pediatric renal transplant recipients weighing less than 30 kg [30]. Conversely, a 2005 analysis by the UNOS revealed higher rates of delayed graft function and acute graft rejection in younger recipients who underwent LDN [29]. However, these subgroup findings should be interpreted cautiously, as they are based on only four predominantly historical retrospective studies and the certainty of evidence was rated as very low. In addition, residual confounding, confounding by indication, donor and recipient selection, and center-specific selection practices may have contributed to the observed differences. Accordingly, these associations should not be interpreted as evidence of a causal disadvantage of LDN in contemporary pediatric kidney transplantation.

Vascular thrombosis is a clinically important early complication in pediatric kidney transplantation and may lead to early graft loss, particularly in small recipients. In the present meta-analysis, no statistically significant difference in vascular thrombosis was observed between ODN and LDN. Given the limited number of available studies and events, the available evidence is insufficient to determine whether donor nephrectomy technique influences the risk of vascular thrombosis in pediatric recipients. Further comparative studies are warranted to clarify this question.

Recipient age at transplantation has been associated with graft outcomes [31,32]. However, the mechanisms underlying the observed subgroup differences in very young recipients remain uncertain. According to previous studies, LDN is associated with a longer warm ischemia time and operation time than ODN [7]. Although a previous study suggested shorter warm ischemia times with ODN, our pooled analysis did not demonstrate a statistically significant difference. Therefore, the observed differences in very young recipients may be attributable to factors beyond ischemia time alone, including learning-curve effects, donor hemodynamics, pneumoperitoneum-related alterations in renal perfusion, or center-specific expertise [7]. We have previously shown that surpassing the learning curve can reduce warm ischemia and operation times in LDN [33], and these outcomes could be improved further by robotic-assisted donor nephrectomy. Surgical techniques and skills need to be advanced to meet the unique size and needs of younger recipients. Therefore, medical centers with limited experience in pediatric transplants might face higher instances of complications such as delayed graft function and acute graft rejection [3,21].

Another possible explanation for differences in early graft outcomes may relate to the physiological effects of pneumoperitoneum during laparoscopic donor nephrectomy. Pneumoperitoneum can transiently reduce renal blood flow in the donor kidney before graft retrieval [34]. However, we agree that this effect occurs in the donor rather than the recipient, and it remains unclear whether such transient alterations in renal perfusion translate into clinically relevant effects after transplantation. Therefore, this mechanism should be interpreted cautiously and considered a hypothetical contributing factor rather than a proven explanation for differences in recipient outcomes.

The substantial contribution of the UNOS dataset to the subgroup analysis warrants careful interpretation. The period analyzed (2000–2002) coincided with the rapid adoption of LDN and a documented learning curve. It is therefore plausible that early technical experience rather than the surgical approach itself influenced outcomes in younger recipients. Advances in laparoscopic instrumentation, surgical expertise, graft extraction and handling, and perioperative management may have reduced warm ischemia time and other technical limitations associated with early LDN practice. Contemporary robotic-assisted approaches may further mitigate some of these limitations. In this context, Walther et al. reported comparable graft outcomes following laparoscopic donor nephrectomy in pediatric recipients and did not identify the surgical approach as an independent predictor of adverse outcomes. These findings suggest that, particularly in experienced centers, LDN may achieve outcomes similar to ODN, especially in younger pediatric patients [35].

A recent systematic review by Dagnæs-Hansen et al. evaluating different surgical approaches for living donor nephrectomy in predominantly adult populations similarly concluded that no single surgical approach appears clearly superior overall. The authors reported generally low complication rates and no donor mortality but emphasized substantial heterogeneity in complication reporting, with only one-third of studies using the Clavien–Dindo classification. They further highlighted that the choice of donor nephrectomy technique depends on multiple factors, including warm ischemia time, blood loss, surgeon expertise, and institutional experience. These findings support the interpretation that the donor nephrectomy approach should not be evaluated in isolation but rather within the context of center-specific expertise and standardized outcome reporting [36].

From a clinical perspective, the choice between ODN and LDN should be individualized according to recipient characteristics, donor anatomy, and center expertise. Given that most comparative evidence originates from the early adoption era of LDN, outcomes from older series may not reflect current practice in contemporary high-volume centers with greater minimally invasive surgical experience and standardized perioperative management.

Limitations of this study include the retrospective nature of all included studies, the absence of randomized trials, limited reporting of donor characteristics, and the lack of detailed information on immunosuppressive treatments and the causes of kidney failure. An important limitation is that most of the available comparative evidence originates from the early adoption era of LDN, with a substantial proportion of the data derived from the early 2000s. Although our literature search was performed up to the end of 2025, no contemporary comparative studies between LDN and ODN were identified. Substantial statistical heterogeneity was observed for several outcomes, likely reflecting differences in recipient and donor characteristics, donor selection, surgical expertise, perioperative management, study era, and definitions of clinical endpoints across the included studies. This heterogeneity reduces the precision and robustness of the pooled estimates and further limits their generalizability to contemporary pediatric transplant practice. Accordingly, pooled effect estimates, particularly for outcomes with high heterogeneity, should be interpreted cautiously. Meta-regression and additional subgroup analyses according to study era, center volume, donor age, or laparoscopic learning curve were not performed because of the limited number of studies contributing to individual outcomes and the inconsistent reporting of these study-level characteristics. Therefore, potential sources of heterogeneity could not be formally explored, and the pooled estimates should be interpreted in the context of these clinical and methodological differences. Moreover, outcomes reported in earlier studies may have been influenced by the learning curve associated with the initial adoption of LDN. Furthermore, definitions of delayed graft function and acute graft rejection were not uniform across the included studies. This variability may have contributed to clinical heterogeneity and may affect the comparability and precision of the pooled estimates. Because sufficiently detailed data were not consistently available across studies, sensitivity analyses based on harmonized outcome definitions could not be reliably performed.

Importantly, the precise role of ODN in the modern era of pediatric kidney transplantation remains uncertain, particularly in very young recipients. The currently available comparative evidence is derived predominantly from studies performed during the early adoption phase of laparoscopic donor nephrectomy (LDN) and therefore may not fully reflect outcomes achievable with contemporary minimally invasive and robotic-assisted techniques. In addition, the optimal donor nephrectomy approach is likely highly context-dependent and influenced by multiple factors, including recipient age and size, donor anatomy, center volume, surgical expertise, and multidisciplinary perioperative management. Consequently, conclusions regarding superiority of one technique over another should be interpreted cautiously. Future research should therefore focus on contemporary multicenter studies and large registry-based analyses evaluating modern laparoscopic and robotic-assisted donor nephrectomy, with standardized reporting of surgical and perioperative outcomes. Dedicated analyses of very young recipients, particularly those aged ≤5 years, are needed to determine whether the subgroup differences observed in historical studies persist in contemporary practice.

As LDN has become the standard approach in many transplant centers—and robotic-assisted techniques have further refined minimally invasive donor surgery—prospective randomized comparisons with ODN may be difficult to conduct in contemporary practice. Many technical limitations associated with laparoscopic experience have since been addressed through advances in surgical expertise, perioperative management, and robotic-assisted approaches.

## 5. Conclusions

In conclusion, the available evidence suggests broadly comparable outcomes between ODN and LDN in pediatric KTx. Although subgroup analyses demonstrated lower rates of delayed graft function and acute graft rejection following ODN in recipients aged five years and younger, these findings are based on a limited number of predominantly historical studies with very-low-certainty evidence and should therefore be interpreted cautiously. Given the widespread establishment of minimally invasive and robotic-assisted donor nephrectomy, future investigations should focus on contemporary, high-volume center experiences with detailed reporting of surgical technique, perioperative management, and recipient age stratification to clarify outcomes in very young pediatric recipients.

## Figures and Tables

**Figure 1:**
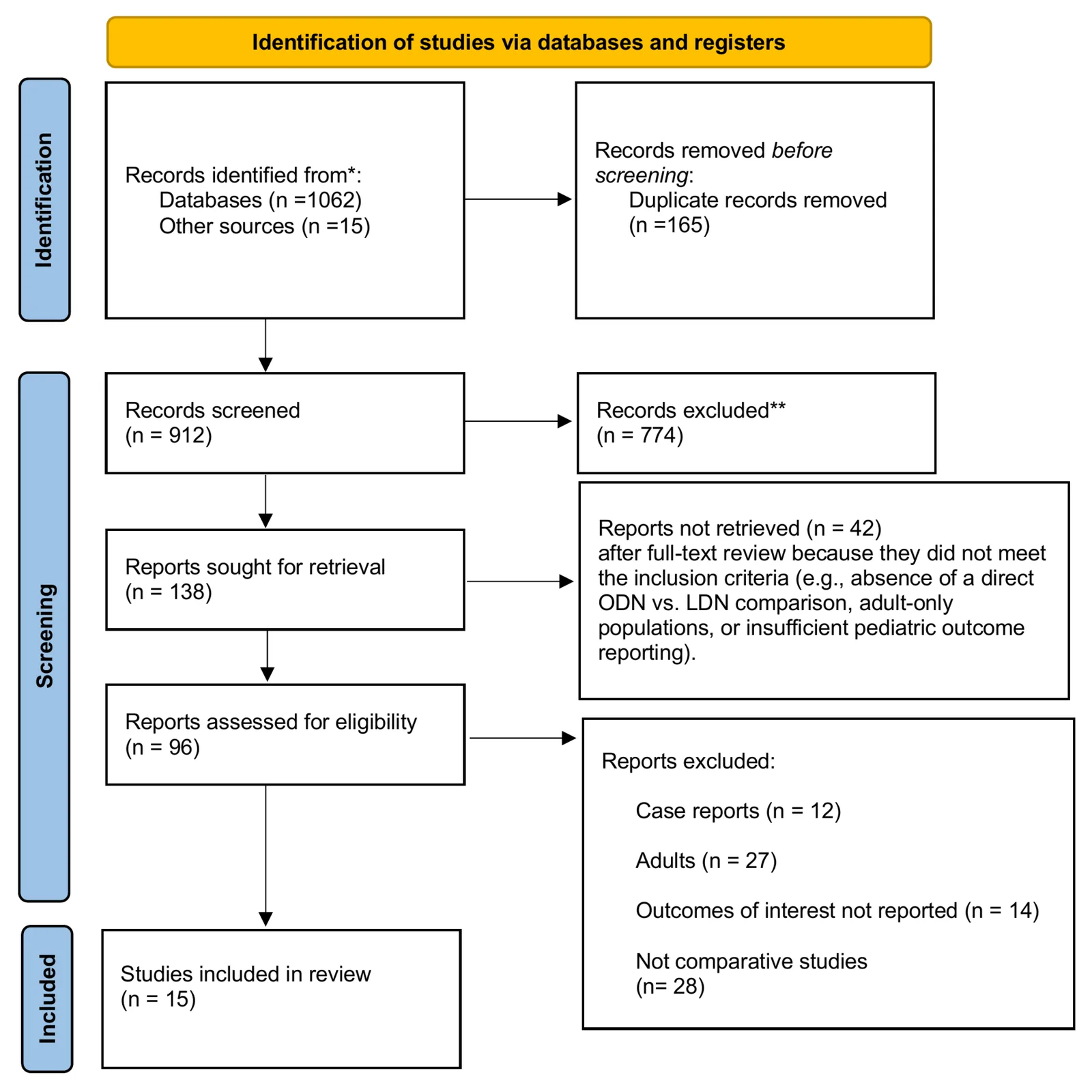
PRISMA flow chart. * Databases including Medline through PubMed, Web of Sciences; Other sources were added through manually search in google scholar and google. ** No Automation was used and all records were excluded by a human.

**Figure 2:**
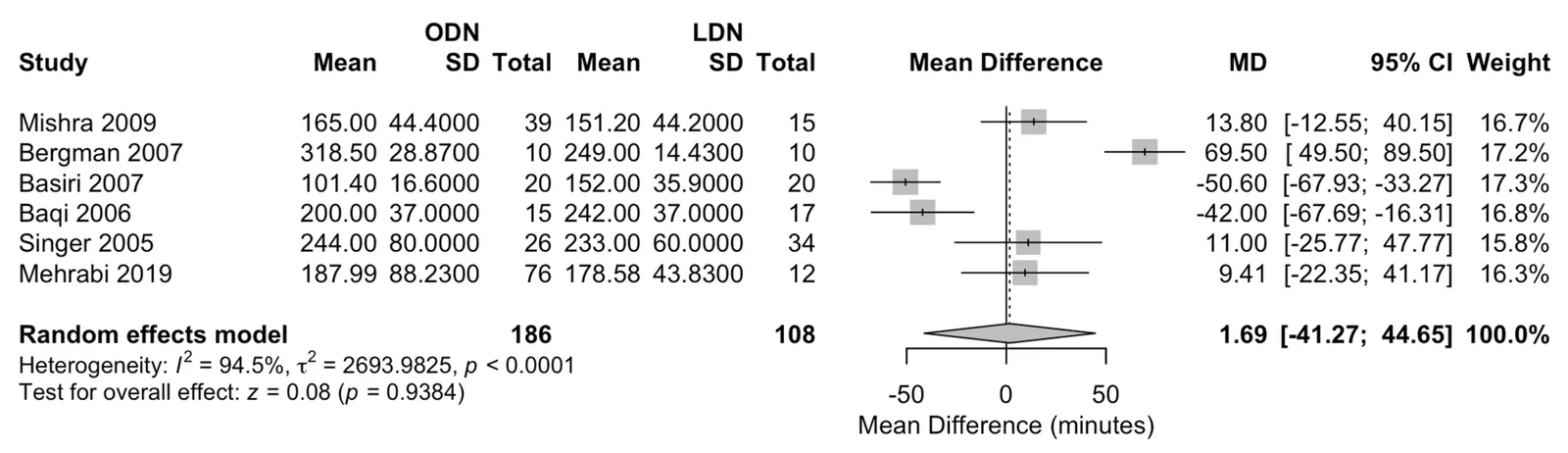
Forest plot showing the comparison of operation time between ODN and LDN in donors using a random-effects model [9,13,21,22,23,24].

**Figure 3:**
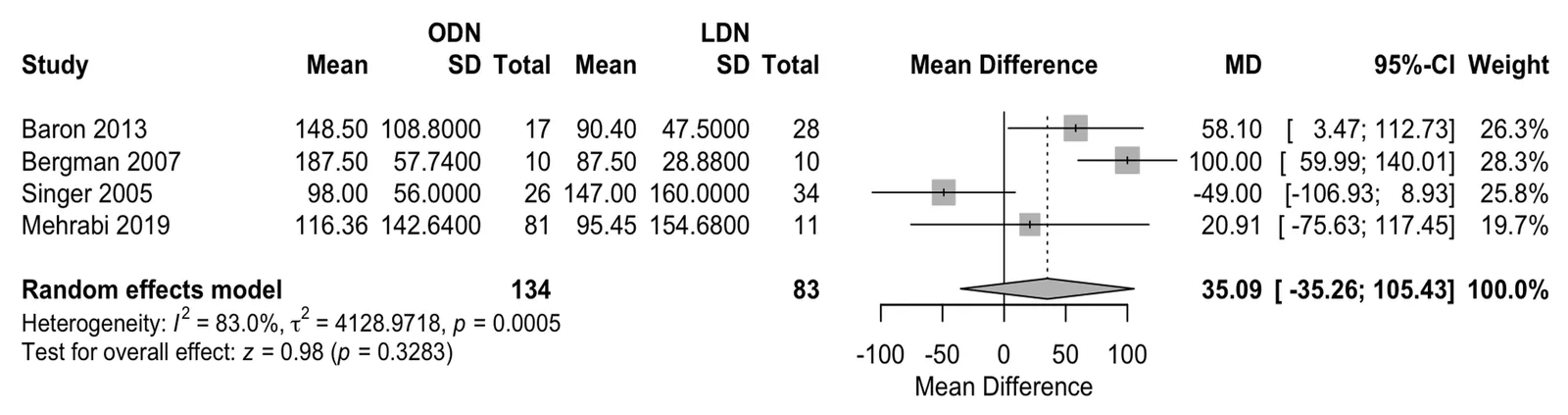
Forest plot showing the comparison of blood loss between ODN and LDN in donors using a random-effects model [13,21,23,27].

**Figure 4:**
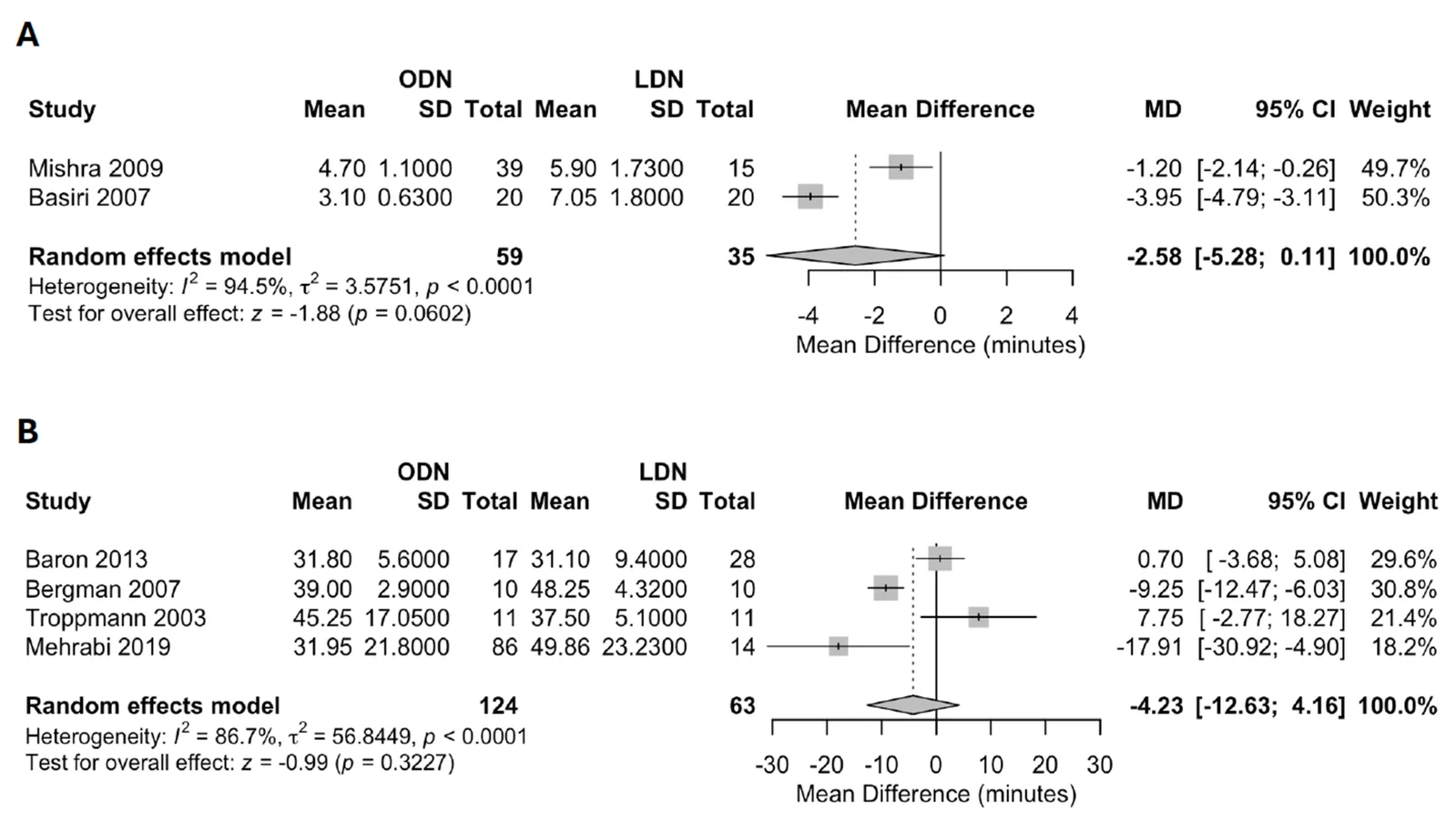
Forest plot showing the comparison of warm ischemia time between ODN and LDN: (**A**) Donor warm ischemia time. (**B**) Recipient warm ischemia using a random-effects model [9,13,18,23,24,27].

**Figure 5:**
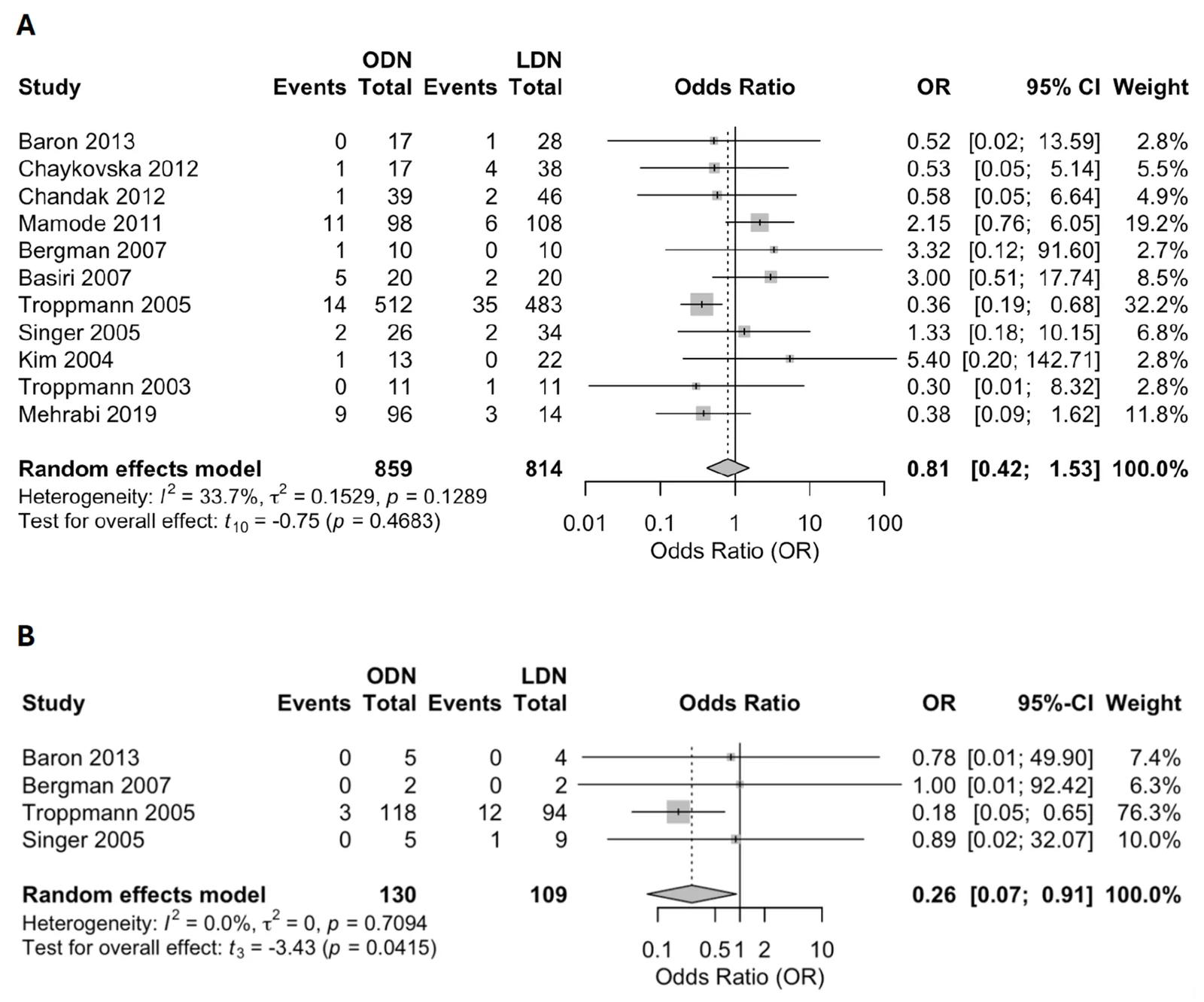
(**A**) Forest plot showing the comparison of delayed graft function between ODN and LDN in pediatric recipients using a random-effects model [3,9,10,13,18,20,21,23,25,26,27]. (**B**) Subgroup analysis of delayed graft function in recipients younger than 5 years. [3,21,23,27].

**Figure 6:**
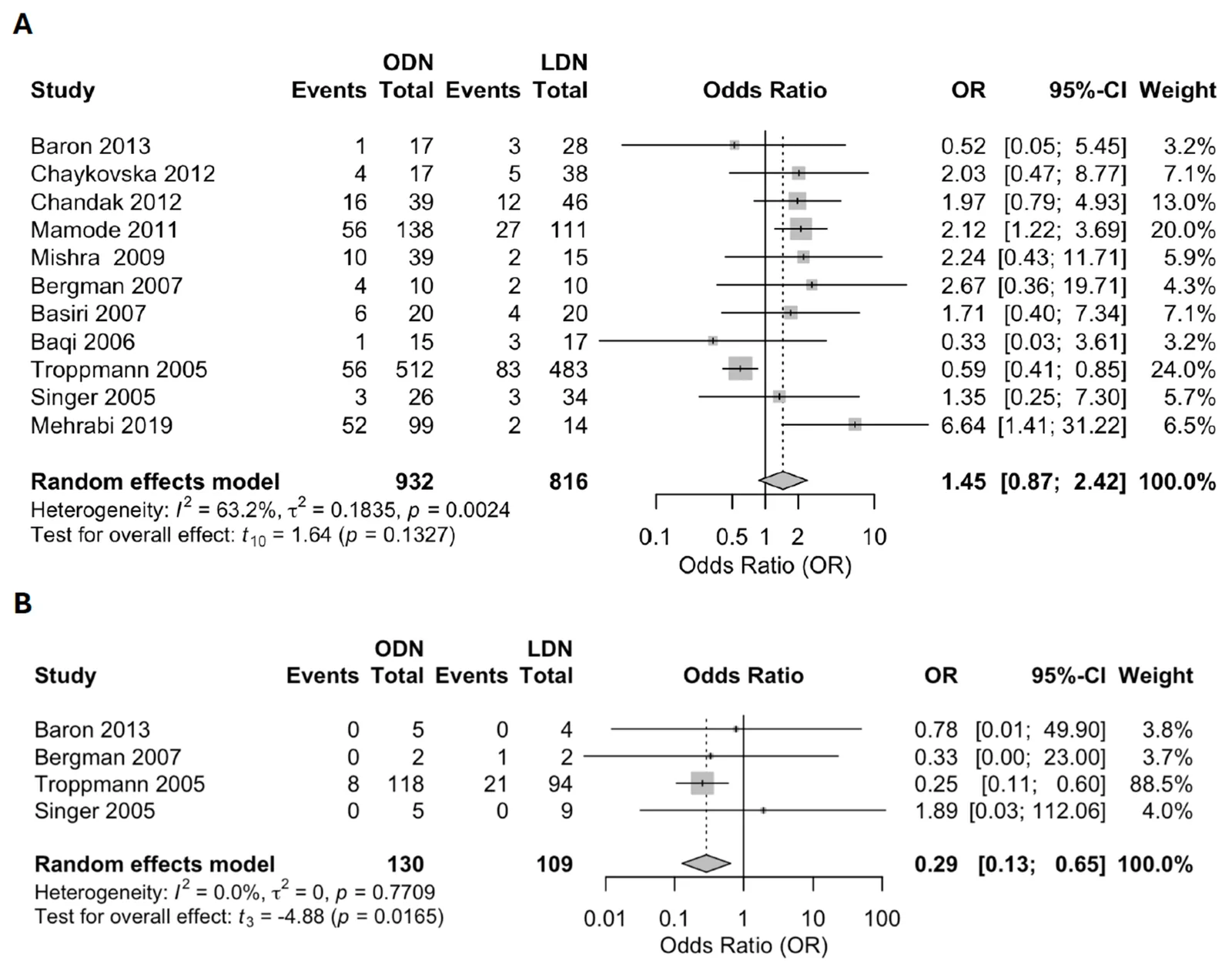
(**A**) Forest plot showing the comparison of acute graft rejection between ODN and LDN in pediatric recipients using a random-effects model [3,9,10,13,21,22,23,24,25,26,27]. (**B**) Subgroup analysis of acute graft rejection in recipients younger than 5 years [3,21,23,27].

**Figure 7:**
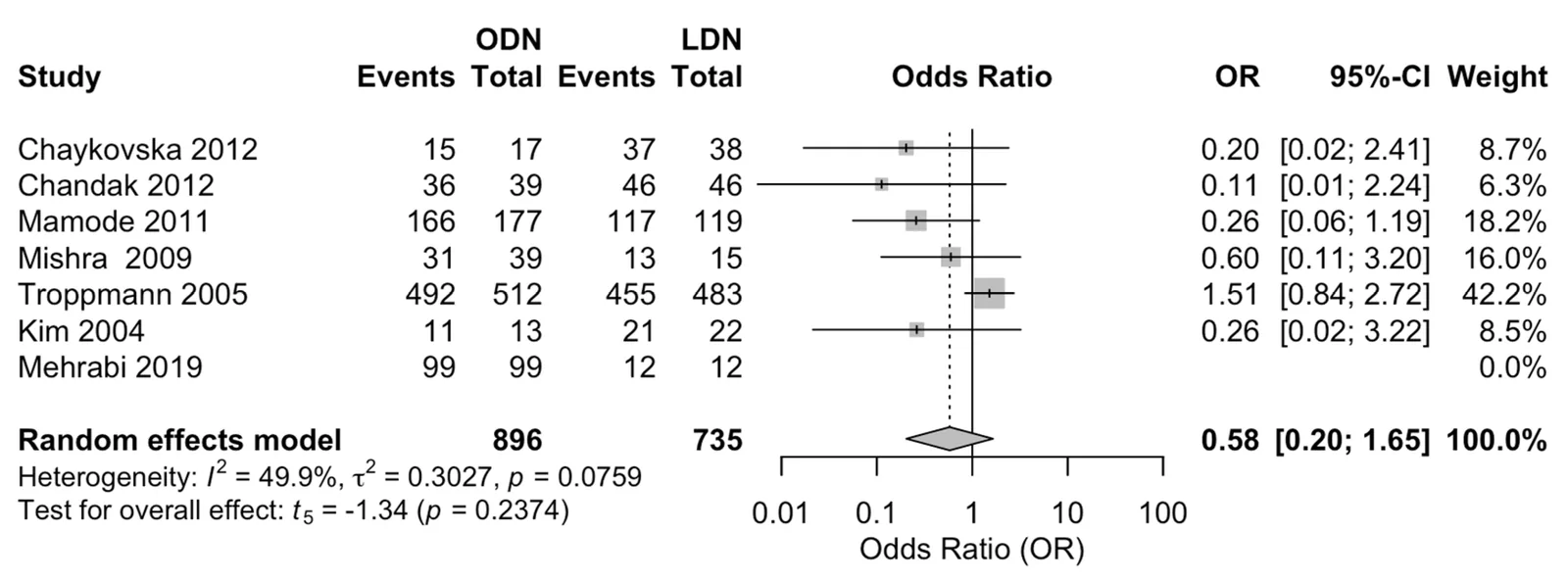
Forest plot showing the comparison of one-year graft survival of between ODN and LDN in pediatric recipients using a random-effects model [3,10,13,20,24,25,26].

**Figure 8:**
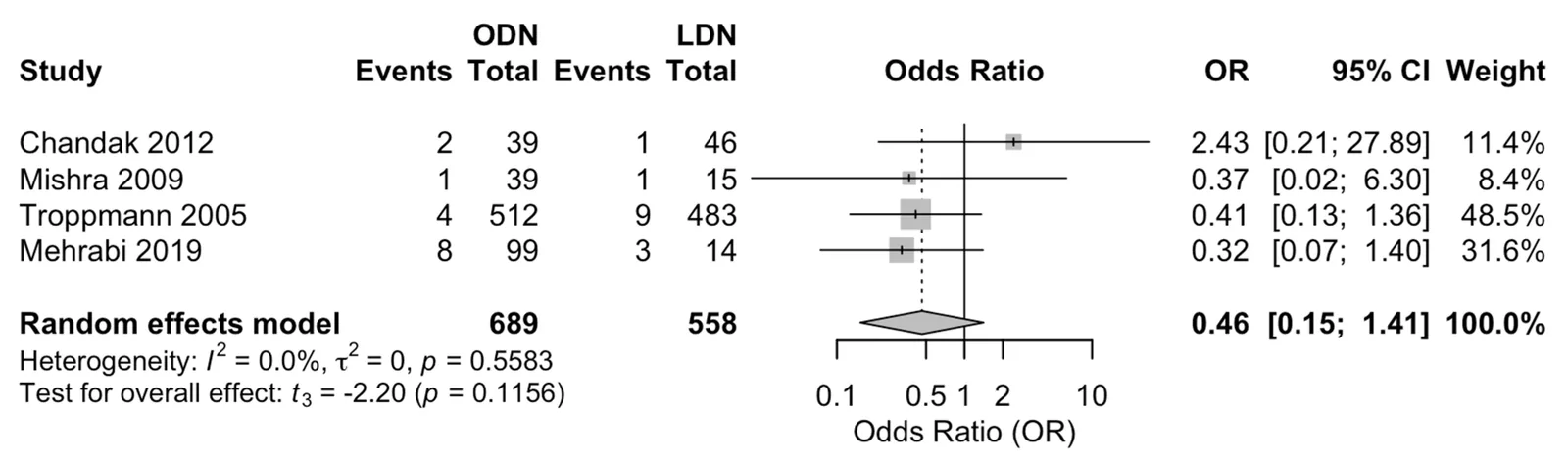
Forest plot showing the comparison of vascular thrombosis between ODN and LDN in pediatric recipients using a random-effects model [3,10,13,24].

**Table 1:** Characteristic of included studies.

				Recipient														Donor						
Author	Type of Study	Country	Year	Group	Number of Patients	Age (Years) Mean ± SD or Range	Gender (Female/Male)	Height (cm) Mean ± SD or Range	Weight (kg) Mean ± SD or Range	BMI Mean ± SD or Range	ASA (II/III)	Re-Transplant (%)	Pre-Emptive Transplant (%)	Primary Urological Disease (%)	Preoperative Dialysis (%)	Urine Volume (mL) in First 24 h Mean ± SD	Follow-Up Mean ± SD	Gender (Female/Male)	Age (Years) Mean ± SD or Range	Height (cm) Mean ± SD or Range	Weight (kg) Mean ± SD or Range	BMI Mean ± SD or Range	VAPS Mean ± SD or Range	Biologically Related/Unrelated
Kayler et al [17]	Retrospective	USA	2002	LDN	13	9.8 (1.6–16)	5/6	NA	39 ± 21 (9–58)	NA	NA	NA	NA	8	6	NA	NA	NA	NA	NA	NA	NA	NA	NA
				ODN	13	10.8 (1–17)	6/7	NA	33 ± 18 (11–68)	NA	NA	NA	NA		5	NA	NA	NA	NA	NA	NA	NA	NA	NA
Troppmann et al [18]	Retrospective	USA	2003	LDN	11	11.9 (1.6–17.7)	4/7	138 (68–170)	34 (7–74)	NA	NA	0	36.36	18.00	NA	NA	NA	5/6	37 (19–48)	NA	NA	NA	NA	9/2
				ODN	11	12.6 (1.2–17.3)	2/9	135 (68–160)	26 (8–59)	NA	NA	9.09	45.45	36.00	NA	NA	NA	7/4	34 (18–44)	NA	NA	NA	NA	11/0
Abrahams et al. [19]	Retrospective	USA	2004	LDN	20	13 (1.7–18)	8/12	NA	43.2 (11–84)	NA	NA	NA	NA	NA	NA	NA	13.6	9/11	40 (26–52)	NA	84.8 (60–107)	NA	NA	NA
				ODN	26			NA		NA	NA	NA	NA	NA	NA	NA				NA		NA	NA	NA
Kim et al [20]	Retrospective	USA	2004	LDN	22	37 ± 7	10/12	NA	NA	NA	NA	18.80	NA	NA	NA	NA	12	10/12	37 ± 7	NA	NA	27.1 (21.6–39.4)	NA	18/4
				ODN	13	35 ± 8	9/4	NA	NA	NA	NA	7.00	NA	NA	NA	NA	12	9/4	35 ± 8	NA	NA	27.9 (17.9–34.7)	NA	12/1
Singer et al [21]	Retrospective	USA	2005	LDN	34	12 ± 6.1	17/17	NA	NA	20 ± 4.1	NA	NA	NA	17.64	NA	NA	16 ± 12	NA	33 ± 8.6	NA	76.9 ± 15.5	NA	NA	34/34
				ODN	26	12 ± 6.5	9/17	NA	NA	21 ± 7.1	NA	NA	NA	34.61	NA	NA	26 ± 12	NA	35 ± 10.2	NA	73.9 ± 12.8	NA	NA	26/26
Troppmann et al. [3]	Retrospective	USA	2005	LDN (0–5 years)	94	2.4 ± 1.4	29/71	85 ± 20	14.3 ± 11.2	NA	NA	2.00	24.00	NA	76.00	NA	NA	(62/38)	33.0 ± 8.3	170 ± 9	74.3 ± 15.9	NA	NA	95/5
				LDN (6–18 years)	389	13.5 ± 3.5	42/58	149 ± 23	49.0 ± 21.6	NA	NA	8.00	40.00	NA	60.00	NA	NA	(54/46)	37.9 ± 8.7	170 ± 11	78.1 ± 17.6	NA	NA	89/11
				ODN (0–5 years)	118	2.2 ± 1.5	37/63	86 ± 18	13.7 ± 6.7	NA	NA	3.00	31.00	NA	69.00	NA	NA	(54/46)	32.9 ± 7.9	171 ± 10	77.5 ± 166	NA	NA	7/3
				ODN (6–18 years)	394	13.7 ± 3.5	41/59	151 ± 21	49.5 ± 20.4	NA	NA	6.00	35.00	NA	65.00	NA	NA	(55/45)	38.8 ± 8.3	169 ± 11	79.3 ± 15.7	NA	NA	93/7
Baqi et al [22]	Retrospective	USA	2006	LDN	17	11.25 ± 3.75	7/10	NA	41.12 ± 13.87	NA	NA	NA	47.00	41.00	53.00	NA	NA	NA	NA	NA	NA	NA	NA	NA
				ODN	15	9.20 ± 4.96	4/11	NA	41.22 ± 28.05	NA	NA	NA	33.00	53.00	67.00	NA	NA	NA	NA	NA	NA	NA	NA	NA
Bergman S et al. [23]	Retrospective	Canada	2007	LDN	10	9.83 ± 5.19	5/5	1.29 ± 0.13	32.19 ± 9.10	NA	2/8	NA	10.00	NA	90.00	NA	22.7 ± 5.17	NA	NA	NA	NA	NA	NA	NA
				ODN	10	10.5 ± 6.67	2/8	1.31 ± 0.51	36.37 ± 9.85	NA	3/7	NA	10.00	NA	90.00	NA	34.07 ± 19.87	NA	NA	NA	NA	NA	NA	NA
Basiri et al [9]	Retrospective	Iran	2007	LDN	20	12.1 ± 3.4	9/11	129.8 ± 17.8	31 ± 11.4	NA	NA	NA	NA	NA	NA	11,447.02 ± 4399.05	211.7 ± 79.39	NA	NA	NA	NA	NA	5.6 ± 1.1	NA
				ODN	20	12 ± 3.8	8/12	137.4 ± 18.9	34 ± 18	NA	NA	NA	NA	NA	NA	9836.84 ± 4768.95	195.9 ± 79.40	NA	NA	NA	NA	NA	5.5 ± 1.6	NA
Mishra et al [24]	Retrospective	India	2009	LDN	15	14.8 (5–18)	4/11	NA	40.4 ± 16.9	NA	NA	NA	66.67	20	66.67	NA	12	9/6	42.0 (27–56)	64.4 ± 8.9	NA	NA	NA	3/12
				ODN	39	13.9 (8–18)	33/6	NA	31.6 ± 9.4	NA	NA	NA	2.56	23.07	97.43	NA	12	27/12	37.7 (19–57)	59.2 ± 6.8	NA	NA	NA	27/12
Chandak et al [10]	Retrospective	UK	2012	LDN	46	9.8 ± 5.04	25/21	NA	32.8 (17)	NA	NA	NA	41.30	43.47	58.69	NA	13	NA	NA	NA	NA	NA	NA	NA
				ODN	39	10.4 ± 4.67	25/14	NA	33 (14.85)	NA	NA	NA	20.51	46.15	79.49	NA	26	NA	NA	NA	NA	NA	NA	NA
Mamode et al [25]	Retrospective	UK	2011	LDN	119	10.7 (0.5)	75/119	NA	NA	18.9 (0.4)	NA	NA	NA	NA	NA	NA	32	66/119 (55.5)	NA	NA	NA	25.8 (0.4)	NA	NA
				ODN	178	10.7 (0.4)	111/178	NA	NA	19.8 (0.5)	NA	NA	NA	NA	NA	NA	47	86/178 (48.3%)	NA	NA	NA	26.6 (0.4)	NA	NA
Chaykovska et al. [26]	Retrospective	Germany	2012	LDN	38	15 (3–18)	15/23	NA	NA	NA	NA	NA	23.68	21.05	42.0 (27–56)	NA	64	22/16	41 (24–67)	NA	NA	NA	NA	NA
				ODN	17	14 (5–18)	8/9	NA	NA	NA	NA	NA	23.52	11.76	76.47	NA	137	15/2	38 (24–48)	NA	NA	NA	NA	NA
Baron et al [27]	Retrospective	USA	2013	LDN	28	12.8 ± 4.8	14/14	NA	NA	19.78 ± 3.97	NA	NA	NA	28.57	NA	NA	12	NA	NA	NA	NA	NA	NA	NA
				ODN	17	10.4 ± 6.6	9/8	NA	NA	19.42 ± 5.0	NA	NA	NA	29.41	NA	NA	12	NA	NA	NA	NA	NA	NA	NA
Mehrabi et al. [13]	Retrospective	Germany	2019	LDN	14	11.20 ± 4.48	6/8	1.41 ± 0.27	38.18 ± 21.81	17.40 ± 4.69	NA	NA	57.14	NA	NA	NA	60	NA	NA	NA	NA	NA	NA	NA
				ODN	100	10.40 ± 5.04	35/65	1.28 ± 0.31	31.63 ± 17.96	17.43 ± 3.26	NA	NA	71.57	NA	NA	NA	60	NA	NA	NA	NA	NA	NA	NA

ODN, open donor nephrectomy; LDN, laparoscopic donor nephrectomy; BMI, body mass index; ASA, American Society of Anesthesiologists physical status classification; VAPS, visual analog pain scale; NA, not available; SD, standard deviation; USA, United States of America; UK, United Kingdom.

## Data Availability

The data used in this systematic review and meta-analysis can be available through request from the corresponding author.

## Supplementary Materials

The following supporting information can be downloaded at: https://www.mdpi.com/article/10.3390/jcm15197484/s1, Text S1: Complete database-specific search strategies; Table S1: Quality assessment of the included studies using the ROBINS-I tool; Table S2: Grades of Recommendation, Assessment, Development, and Evaluation (GRADE) assessment for outcomes of interest; Table S3: Meta-analysis results and quality of evidence for outcomes; Table S4: Study-level sample sizes and raw event data for post-transplant outcomes included in the meta-analysis; Table S5: Prisma 2020 Checklist.

## Abbreviations

The following abbreviations are used in this manuscript:

ODN	Open Donor Nephrectomy
LDN	Laparoscopic Donor Nephrectomy
KTx	Kidney Transplantation
ESKD	End-Stage Kidney Disease
