# Supplementary material for: Open Versus Laparoscopic Donor Nephrectomy in Pediatric Kidney Transplantation: A Systematic Review and Meta-Analysis
Source: J Clin Med. 2026 Sep 26;15(19):7484. doi: 10.3390/jcm15197484 (PMC13666085; doi:10.3390/jcm15197484)
Supplement: Supplementary file 1 [file jcm-15-07484-s001.zip › jcm-4478753-supplementary.pdf]

Supplementary Materials:

Supplementary Text S1. Complete database-specific search strategies

The following database-specific search strategies were used to conduct the systematic literature search in MEDLINE via PubMed and Web of Science Core Collection, respectively. The search covered records published through 31 December 2025.

MEDLINE via PubMed:

(("kidney*"[All Fields] OR "renal*"[All Fields]) AND ("explant*"[All Fields] OR "harvest*"[All Fields] OR "procurement"[All Fields] OR "retrieval*"[All Fields] OR "nephrectom*"[All Fields])) AND ("donor*"[All Fields] OR "donation"[All Fields]) AND ("robot*"[All Fields] OR "da vinci*"[All Fields] OR "davinci*"[All Fields] OR "minimal invasive"[All Fields] OR "minimally invasive"[All Fields] OR "laparoscop*"[All Fields] OR "open"[All Fields] OR "surg*"[All Fields] OR "operation"[All Fields]) AND ("pediatr*"[All Fields] OR "paediatr*"[All Fields] OR "child*"[All Fields] OR "infant*"[All Fields] OR "juvenile*"[All Fields]).

Web of Science Core Collection:

TS=((kidney* OR renal*) AND (explant* OR harvest* OR procurement OR retrieval* OR nephrectom*) AND (donor* OR donation) AND (robot* OR "da vinci*" OR "davinci*" OR "minimal invasive" OR "minimally invasive" OR laparoscop* OR open OR surg* OR operation) AND (pediatr* OR paediatr* OR child* OR infant* OR juvenile*))

Supplementary Table S1: Quality assessment of the included studies using the ROBINS-I tool.

	Risk of bias							
**Study**	**Bias due to confounding**	**Bias in selection of participants into the study**	**Bias in classification of interventions**	**Bias due to deviations from intended interventions**	**Bias due to missing data**	**Bias in measurement of outcomes**	**Bias in selection of the reported result**	**ROBINS-I (overall)**
Kayler et al. (2002)	Moderate	Moderate	Low	Moderate	Low	Serious	Serious	Serious
Troppmann et al. (2003)	Low	Moderate	Low	Low	Low	Low	Low	Moderate
Abrahams et al. (2004)	Moderate	Moderate	Low	Moderate	Low	Serious	Serious	Serious
Kim et al. (2004)	Moderate	Moderate	Low	Moderate	Low	Moderate	Moderate	Moderate
Singer et al. (2005)	Low	Moderate	Low	Moderate	Low	Low	Moderate	Moderate
Troppmann et al. (2005)	Low	Moderate	Low	Moderate	Low	Low	Low	Moderate
Baqi et al. (2006)	Moderate	Moderate	Low	Moderate	Low	Low	Low	Moderate
Bergman et al. (2007)	Low	Moderate	Low	Moderate	Low	Serious	Moderate	Serious
Basiri et al. (2007)	Low	Low	Low	Low	Low	Serious	Serious	Serious

Mishra et al. (2009)	Low	Moderate	Low	Moderate	Low	Moderate	Serious	Serious
Chandak et al. (2012)	Low	Moderate	Low	Moderate	Low	Moderate	Moderate	Moderate
Mamode et al. (2011)	Low	Moderate	Low	Moderate	Low	Low	Low	Moderate
Chaykovska et al. (2012)	Low	Moderate	Low	Moderate	Serious	Low	Moderate	Serious
Baron et al. (2013)	Low	Moderate	Low	Moderate	Low	Low	Low	Moderate
Mehrabi et al. (2019)	Low	Low	Low	Moderate	Low	Low	Low	Moderate

Supplementary Table S2: Grades of Recommendation, Assessment, Development, and Evaluation (GRADE) assessment for outcomes of interest

Comparison	Studies	GRADE							
		**Risk of bias**	**Inconsistency**	**Indirectness**	**Imprecision**	**Publication bias**	**Large or moderate effect size**	**Exposure-response gradient**	**Overall quality**
Laparoscopic donor nephrectomy vs. open donor nephrectomy									
Operation time	6	Serious	Serious	Not serious	Serious	Not serious	Not present	Not present	Very low
Estimated blood loss	4	Serious	Serious	Not serious	Serious	Serious	Not present	Not present	Very low
Warm ischemia time in recipients	4	Serious	Not serious	Not serious	Serious	Not serious	Not present	Not present	Very low
Acute graft rejection	11	Very serious	Not serious	Not serious	Serious	Not serious	Not present	Not present	Very low
Delayed graft function	11	Serious	Not serious	Serious	Serious	Not serious	Not present	Not present	Very low
One-year graft survival	7	Very serious	Serious	Not serious	Serious	Serious	Not present	Not present	Very low
Vacular thrombosis	4	Serious	Not serious	Not serious	Serious	Not serious	Not present	Not present	Very low

Supplementary Table S3: Meta-analysis results and quality of evidence for outcomes

**Outcome**	**Number of studies**	**Pooled relative effect (95% CI)**	**Statistical heterogeneity (%); *P*-value**	**Quality of evidence (GRADE)**
Open donor nephrectomy vs. laparoscopic donor nephrectomy				
Operation time	6	MD = 1.69 (−41.27 to 44.65)	94%; *P* <0.0001	Very low
Estimated blood loss	4	MD = 35.09 (−35.26 to 105.43)	83%; *P* <0.001	Very low
Warm ischemia time in recipeints	4	MD = −4.23 (−12.63 to 4.16)	86%; *P* < 0.0001	Very low
Acute graft rejection	11	OR = 1.45 (0.87 to 2.42)	63%; *P* = 0.0024	Very low
Delayed graft function	11	OR = 0.81 (0.42 to 1.53)	33%; *P* = 0.12	Very low

One-year graft survival	7	OR = 0.58 (0.20 to 1.65)	50%; $P = 0.07$	Very low
Vacular thrombosis	4	OR, 0.46 (0.15 to 1.41)	0%; $P = 0.55$	Very low

OR: odds ratio; MD: mean difference

Supplementary Table S4: Study-level sample sizes and raw event data for post-transplant outcomes included in the meta-analysis

Author	Type of study	Year	Group	Number of patients	Acute graft rejection	Delayed graft function	One-year graft survival	Vascular thrombosis
Kayler et al [17]	Retrospective	2002	LDN	13	NA	NA	NA	NA
			ODN	13	NA	NA	NA	NA
Troppmann et al [18]	Retrospective	2003	LDN	11	NA	1	NA	NA
			ODN	11	NA	0	NA	NA
Abrahams et al [19]	Retrospective	2004	LDN	20	NA	NA	NA	NA
			ODN	26	NA	NA	NA	NA
Kim et al [20]	Retrospective	2004	LDN	22	NA	0	21	NA
			ODN	13	NA	1	11	NA
Singer et al [21]	Retrospective	2005	LDN	34	3	2	NA	NA
			ODN	26	3	2	NA	NA
Troppmann et al [3]	Retrospective	2005	LDN (0-5 years)	94	21	12	455/483	9/483
			LDN (6-18 years)	389	62	23		
			ODN (0-5 years)	118	8	3	492	4/512
			ODN (6-18 years)	394	48	11		
Baqi et al [22]	Retrospective	2006	LDN	17	3	NA	NA	NA
			ODN	15	1	NA	NA	NA
Bergman S et al [23]	Retrospective	2007	LDN	10	2	0	NA	NA
			ODN	10	4	1	NA	NA
Basiri et al [9]	Retrospective	2007	LDN	20	4	2	NA	NA
			ODN	20	6	5	NA	NA

Mishra et al [24]	Retrospective	2009	LDN	15	2	NA	13	1
			ODN	39	10	NA	31	1
Chandak et al [10]	Retrospective	2011	LDN	46	12	2	46	1
			ODN	39	16	1	36	2
Mamode et al [25]	Retrospective	2011	LDN	119	27/111	6/108	117	NA
			ODN	178	56/138	11/98	166	NA
Chaykovska et al [26]	Retrospective	2012	LDN	38	5	4	NA	NA
			ODN	17	4	1	NA	NA
Baron et al [27]	Retrospective	2013	LDN	28	3	1	NA	NA
			ODN	17	1	0	NA	NA
Mehrabi et al [13]	Retrospective	2019	LDN	14	2	3	12/12	3
			ODN	100	52/99	9/96	99/99	8/99

***Data are presented as number of events or events/number of patients with available outcome data, as reported in the original studies. LDN, laparoscopic donor nephrectomy; ODN, open donor nephrectomy; NA, not available.**

PRISMA 2020 Checklist

PRISMA

Supplementary Table S5: Prisma 2020 Checklist

Section and Topic	Item #	Checklist item	Location where item is reported
**TITLE**			
Title	1	Identify the report as a systematic review.	Title
**ABSTRACT**			
Abstract	2	See the PRISMA 2020 for Abstracts checklist.	Abstract
**INTRODUCTION**			
Rationale	3	Describe the rationale for the review in the context of existing knowledge.	Introduction, paragraphs 1–3
Objectives	4	Provide an explicit statement of the objective(s) or question(s) the review addresses.	Introduction, paragraph 3
**METHODS**			
Eligibility criteria	5	Specify the inclusion and exclusion criteria for the review and how studies were grouped for the syntheses.	Methods, Section 2.1 (Search Strategy and Selection Criteria)
Information sources	6	Specify all databases, registers, websites, organisations, reference lists and other sources searched or consulted to identify studies. Specify the date when each source was last searched or consulted.	Methods, Section 2.1 (requires revision to specify exact final search date for each database)
Search strategy	7	Present the full search strategies for all databases, registers and websites, including any filters and limits used.	Methods, Section 2.1; full database-specific strategies to be added in Supplementary Appendix
Selection process	8	Specify the methods used to decide whether a study met the inclusion criteria of the review, including how many reviewers screened each record and each report retrieved, whether they worked independently, and if applicable, details of automation tools used in the process.	Methods, Section 2.1
Data collection process	9	Specify the methods used to collect data from reports, including how many reviewers collected data from each report, whether they worked independently, any processes for obtaining or confirming data from study investigators, and if applicable, details of automation tools used in the process.	Methods, Section 2.2 (Data Extraction and Risk of Bias)
Data items	10a	List and define all outcomes for which data were sought. Specify whether all results that were compatible with each outcome domain in each study were sought (e.g. for all measures, time points, analyses), and if not, the methods used to decide which results to collect.	Methods, Sections 2.1–2.2
	10b	List and define all other variables for which data were sought (e.g. participant and intervention characteristics, funding sources). Describe any assumptions made about any missing or unclear information.	Methods, Section 2.2; Table 1
Study risk of bias assessment	11	Specify the methods used to assess risk of bias in the included studies, including details of the tool(s) used, how many reviewers assessed each study and whether they worked independently, and if applicable, details of automation tools used in the process.	Methods, Sections 2.2–2.3; Supplementary Table 1
Effect measures	12	Specify for each outcome the effect measure(s) (e.g. risk ratio, mean difference) used in the synthesis or presentation of results.	Methods, Section 2.4 (Statistical Analysis)
Synthesis methods	13a	Describe the processes used to decide which studies were eligible for each synthesis (e.g. tabulating the study intervention characteristics and comparing against the planned groups for each synthesis (item #5)).	Methods, Sections 2.1–2.2

Section and Topic	Item #	Checklist item	Location where item is reported
	13b	Describe any methods required to prepare the data for presentation or synthesis, such as handling of missing summary statistics, or data conversions.	Not explicitly reported
	13c	Describe any methods used to tabulate or visually display results of individual studies and syntheses.	Methods, Section 2.4; Figures 2–9 and Table 1
	13d	Describe any methods used to synthesize results and provide a rationale for the choice(s). If meta-analysis was performed, describe the model(s), method(s) to identify the presence and extent of statistical heterogeneity, and software package(s) used.	Methods, Section 2.4 (Statistical Analysis)
	13e	Describe any methods used to explore possible causes of heterogeneity among study results (e.g. subgroup analysis, meta-regression).	Methods, Sections 2.1 and 2.4 (prespecified age subgroup; heterogeneity assessment)
	13f	Describe any sensitivity analyses conducted to assess robustness of the synthesized results.	Methods, Section 2.4 (leave-one-out sensitivity analysis)
Reporting bias assessment	14	Describe any methods used to assess risk of bias due to missing results in a synthesis (arising from reporting biases).	Not reported
Certainty assessment	15	Describe any methods used to assess certainty (or confidence) in the body of evidence for an outcome.	Methods, Section 2.3 (GRADE)
**RESULTS**			
Study selection	16a	Describe the results of the search and selection process, from the number of records identified in the search to the number of studies included in the review, ideally using a flow diagram.	Results, Section 3.1; Figure 1 (PRISMA flow diagram)
	16b	Cite studies that might appear to meet the inclusion criteria, but which were excluded, and explain why they were excluded.	Results, Section 3.1; Figure 1
Study characteristics	17	Cite each included study and present its characteristics.	Results, Section 3.1; Table 1; References 17–27 and 13
Risk of bias in studies	18	Present assessments of risk of bias for each included study.	Results, Section 3.2; Supplementary Table 1
Results of individual studies	19	For all outcomes, present, for each study: (a) summary statistics for each group (where appropriate) and (b) an effect estimate and its precision (e.g. confidence/credible interval), ideally using structured tables or plots.	Results, Sections 3.3–3.4; Figures 2–9
Results of syntheses	20a	For each synthesis, briefly summarise the characteristics and risk of bias among contributing studies.	Results, Sections 3.1–3.2; Table 1; Supplementary Table 1
	20b	Present results of all statistical syntheses conducted. If meta-analysis was done, present for each the summary estimate and its precision (e.g. confidence/credible interval) and measures of statistical heterogeneity. If comparing groups, describe the direction of the effect.	Results, Sections 3.3–3.4; Figures 2–9; Supplementary Table 3
	20c	Present results of all investigations of possible causes of heterogeneity among study results.	Results, Sections 3.3–3.4 (subgroup and heterogeneity results)
	20d	Present results of all sensitivity analyses conducted to assess the robustness of the synthesized results.	Results, Sections 3.3.1–3.3.3 (sensitivity analyses)
Reporting biases	21	Present assessments of risk of bias due to missing results (arising from reporting biases) for each synthesis assessed.	Not reported
Certainty of evidence	22	Present assessments of certainty (or confidence) in the body of evidence for each outcome assessed.	Results, Section 3.2; Supplementary Tables 2–3
**DISCUSSION**			
Discussion	23a	Provide a general interpretation of the results in the context of other evidence.	Discussion, paragraphs 1–8
	23b	Discuss any limitations of the evidence included in the review.	Discussion, Limitations paragraph
	23c	Discuss any limitations of the review processes used.	Not explicitly reported
	23d	Discuss implications of the results for practice, policy, and future research.	Discussion, final two paragraphs; Conclusions

Section and Topic	Item #	Checklist item	Location where item is reported
**OTHER INFORMATION**			
Registration and protocol	24a	Provide registration information for the review, including register name and registration number, or state that the review was not registered.	Methods, opening paragraph (PROSPERO CRD4202450558)
	24b	Indicate where the review protocol can be accessed, or state that a protocol was not prepared.	PROSPERO registration CRD4202450558; protocol access location should be stated explicitly
	24c	Describe and explain any amendments to information provided at registration or in the protocol.	Not explicitly reported
Support	25	Describe sources of financial or non-financial support for the review, and the role of the funders or sponsors in the review.	Funding statement
Competing interests	26	Declare any competing interests of review authors.	Conflicts of Interest statement
Availability of data, code and other materials	27	Report which of the following are publicly available and where they can be found: template data collection forms; data extracted from included studies; data used for all analyses; analytic code; any other materials used in the review.	Data Availability Statement (requires wording clarification regarding availability of data/code/materials)
